# Not Next to You: Peer Rejection, Sociodemographic Characteristics and the Moderating Effects of Classroom Composition

**DOI:** 10.1007/s10964-023-01758-x

**Published:** 2023-03-10

**Authors:** Simon Hjalmarsson, Peter Fallesen, Stephanie Plenty

**Affiliations:** 1 The Swedish Institute for Social Research, Stockholm University, Stockholm, Sweden; 2 ROCKWOOL Foundation, Copenhagen, Denmark; 3 Institute for Futures Studies, Stockholm, Sweden

## Abstract

While a range of sociodemographic characteristics are associated with a greater risk of peer rejection at school, it is currently unclear how key theoretical frameworks explaining rejection apply to such characteristics. This study examines how migration background, gender, household income, parental education and cognitive ability are linked to peer rejection. Building on person-group dissimilarity and social identity theory, the study assesses the moderating role of classroom composition and the extent to which students reject classmates who differ to themselves (i.e., outgroup derogation). Data is drawn from a nationally representative sample of 4215 Swedish eighth grade students (M_age_ = 14.7, SD_age_ = 0.39; 67% of Swedish origin; 51% girls) in 201 classes. While rejection based on migration background, gender, household income and cognitive ability was moderated by the school-class composition, only the rejection of immigrant background students, boys and girls was related to outgroup derogation. Furthermore, Swedish origin students’ outgroup derogation increased as the share of immigrant background students decreased. Addressing social inequalities in rejection may require different strategies depending on sociodemographic characteristic.

## Introduction

Peer rejection hurts—both figuratively and literally. Poor peer relationships undermines students’ school engagement (Juvonen et al., 2019; Wentzel et al., 2021) and psychological well-being (Timeo et al., 2019), and peer rejection risk is not independent of student characteristics. Factors such as immigration background (Plenty & Jonsson, 2017), low economic resources (Hjalmarsson, 2018), low parental education (Knaappila et al., 2018; Nordhagen et al., 2005) and low school performance (Wentzel et al., 2021) are associated with a greater risk of rejection at school. Yet, it is currently unclear how key theoretical frameworks explaining rejection apply to these characteristics. Using a large sample of Swedish eighth grade students (*n* = 4215) from 201 classrooms, this study assesses the role of person-group dissimilarity and social identity theory processes in producing social inequalities in peer rejection for key sociodemographic characteristics and cognitive ability.

Studies informed by person-group dissimilarity theory (Wright et al., 1986) suggest that adolescents are more likely to be rejected by peers when they noticeably differ from context-specific group norms. In turn, social identity theory (Tajfel, 1982; Tajfel & Turner, 1979) suggests that individuals tend to favor those considered ingroup and to distance themselves from those considered outgroup. While both theoretical processes are likely to contribute to the marginalization of students belonging to minority status social groups, evidence is scarce on whether such processes apply across a range of characteristics as well as whether they combine to jointly shape social inequalities in rejection.

Peer relationships are inevitably embedded in the social contexts in which they develop. Social-ecological models posit that the determinants of rejection depend on complex person-context interactions between individuals and their social environment (Hong & Espelage, 2012). Person-group dissimilarity theory (Wright et al., 1986) extends this perspective by arguing that youth who differ from a group’s descriptive norm in some discernible way are at risk of worse peer relationships. Empirical tests of person-group dissimilarity theory have shown that students with personalities, characteristics, or behaviors that noticeably differ from the group norm experience lower acceptance (Boivin et al., 1995; Stormshak et al., 1999), as well as more bullying and aggression from peers (Boele et al., 2017; Kaufman et al., 2022). Consistent with person-group dissimilarity processes, the well-established association between academic achievement and peer relationships (Wentzel et al., 2021) has been found to be moderated by classroom-level ability (e.g., Jonkmann et al., 2009; Palacios et al., 2019).

In relation to sociodemographic factors, the reasoning of person-group dissimilarity theory has predominantly been applied in research on the victimization of immigrant background and ethnic minority youth. This has brought attention to the moderating effects of school or classroom composition (Kuldas et al., 2021), as immigrant background youth have been found to be more likely to be victimized (Plenty & Jonsson, 2017) and to experience greater loneliness (Madsen et al., 2016) in school settings with fewer immigrant background classmates.

Yet, person-group dissimilarity theory is arguably also applicable to other key sociodemographic characteristics, such as gender (Mikami et al., 2010) and socioeconomic status (Bukowski et al., 2020, 2021). For students belonging to sociodemographic minority or lower status groups at the societal level, classrooms with little sociodemographic representation may exacerbate their social vulnerability. However, classrooms with higher sociodemographic representation of low-status or minority groups could shift the social norms to these groups advantage, thereby reducing the likelihood of rejection. Empirically, gender is a key stratifier of friendship preferences (e.g., Gifford-Smith & Brownell, 2003), consistent with gender forming a strong basis for ingroup and outgroup categorization (Maccoby, 1988). School-class gender composition appears related to non-academic outcomes, as a higher share of girls has been linked to fewer in-school injuries (Filser et al., 2022) but also to worse mental health, particularly among boys (Getik & Meier, 2022). However, research on whether school-class gender composition impacts peer relationships is scarce, one exception being a study following the transition from mixed-sex to same-sex education in one U.S. elementary school (Barton & Cohen, 2004). For socioeconomic characteristics (e.g., household income or parental education), students who have few classmates with a similar socioeconomic background have been found to experience greater loneliness, less peer acceptance, and lower school belonging than those with a larger share of similar classmates (Benner & Wang, 2014; Crosnoe, 2009). While theoretically plausible, in empirical terms little is known about the applicability of person-group dissimilarity theory to sociodemographic characteristics beyond migration background and ethnic minority status.

Person-group dissimilarity theory explains who are more likely to experience rejection in different classrooms, but it does not consider the source of rejections. In seeking to understand intergroup relations, social identity theory suggests that individuals intrinsically categorize themselves and others into groups according to a range of characteristics, such as ethnicity, gender, and socioeconomic background. Individuals hold multiple identities according to these categories, which shape self-concept, preferences, and behaviors (Tajfel, 1982; Tajfel & Turner, 1979). Due to an assumed sense of familiarity and to boost self-esteem and maintain a positive view of one’s affiliated groups, intergroup biases develop, with a tendency to favor others perceived as belonging to the “ingroup” rather than those considered members of the “outgroup” (ingroup-favoritism) (e.g., Boda & Neray, 2015).

An intergroup bias that involves negative evaluations, attitudes or behaviors towards outgroup members is known as *outgroup derogation* (Hewstone et al., 2002). As rejection entails distancing oneself psychologically or behaviorally from another person (Leary, 2015), the study conceptualizes the rejection of classmates with characteristics distinct to oneself as being indicative of outgroup derogation. Drawing on social identity theory, it is thus expected that students reject classmates who differ from themselves on a sociodemographic characteristic more than they reject classmates similar to themselves.

Although person-group dissimilarity and social identity theory have different focuses they are complementary and in combination they may provide a rich understanding of peer rejection. Social identity theory suggests that individuals distance themselves from others perceived as belonging to different social groups (i.e., outgroup derogation towards different-characteristic peers) but does not address the role of classroom composition. Students may experience greater rejection in contexts where their ingroup is less represented because person-group dissimilarity processes lead the situational majority’s outgroup derogation to strengthen. Yet, students could also experience greater rejection in such contexts due to other factors, such as the larger number of outgroup peers simply meaning that there are more outgroup peers available to make rejections. To better understand how classroom composition influences peer rejection, it is thus necessary to assess the role of outgroup derogation in person-group dissimilarity processes.

## Current Study

While a range of sociodemographic characteristics are associated with a greater risk of peer rejection, it is currently unclear how key theoretical frameworks explaining rejection apply to such characteristics. Consequently, this study asks how migration background, gender, household income, and parental education, are linked with peer rejection, thereby assessing the applicability of person-group dissimilarity and social identity theory to a range of key sociodemographic characteristics. Given the important ties between scholastic performance and peer relationships in educational settings, the abovementioned theoretical processes are assessed also for cognitive ability. Based on person-group dissimilarity it was hypothesized that students would receive more rejection nominations in classes where their characteristic was less prevalent (Hypothesis 1). In line with social identity theory, it was hypothesized that students would send more rejection nominations to different-characteristic classmates than same-characteristic classmates (Hypothesis 2). However, by integrating the processes drawn from person-group dissimilarity and social identity theory, it was hypothesized that such outgroup derogation would be stronger in classes where different-characteristic classmates were less prevalent (Hypothesis 3).

## Methods

### Data

Data come from the Children of Immigrants Longitudinal Study in Four European Countries (CILS4EU), a project designed to examine the structural, cultural, and social integration of youth in Europe (Kalter et al., 2014). The current study uses Swedish data from the first wave (winter 2010 and spring 2011) when participants were in the eighth grade (approximately 14–15 years of age).^1^ As eighth graders attend almost all lessons together with their classmates and have typically done so for ~2–4 years (depending on the school), the school-class represents a core social arena for Swedish youth.

Statistics Sweden (the Swedish national statistics agency) collected data in schools across Sweden using a three-step stratified sampling approach. Schools were randomly selected within four strata based on the share of students of immigrant background within the school, oversampling schools with a higher share of students with an immigration background. Two classes within each school were then randomly sampled and then all students in the selected classes were invited to participate (see CILS4EU, 2016). Students completed sociometric nominations, a questionnaire, and ability tests during normal lesson time. The CILS4EU data collection was approved by the Regional Ethics Committee of Stockholm (approval reference number 2010/1557–31/5), and all participating students and their parents provided informed consent.

The base sample consisted of 5699 students, in 251 classes, and 129 schools. The following cases were omitted: a) students who did not participate in the survey (*n* = 674); b) 26 students (including the sociometric nominations they made) deemed to have provided unreliable or implausible responses; c) 38 classes (616 students) where less than 70% of students responded to the peer rejection item, following recommendations to ensure valid sociometric data (e.g., Cillessen & Marks, 2011); d) 13 classes with less than 12 participating students to improve the validity of the rejection measure. The analytical sample thus consists of 4215 students in 201 classrooms and 119 schools (74% of students from the base sample; M_age_ = 14.7, SD_age_ = 0.39; 67% of Swedish origin; 51% girls).

### Measures

#### Peer rejection

Although rejection can be conceptualized in many ways, it generally involves devaluing a relationship with another individual and a desire to increase one’s physical and psychological distance to that individual (Leary, 2015). This study operationalizes peer rejection as the preference for not wanting to sit next to a classmate. The strength of this measure (unlike victimization or dislike nominations) is that it permits a broad perspective, encompassing subtle acts of avoidance to potentially overt hostile relationships.

In the CILS4EU, students were presented with a roster listing the names of all classmates (including non-participating students) and asked a number of sociometric questions. In the first wave, this sociometric part of the survey asked students to nominate from zero to five classmates that they did not want to sit next to.^2^ On average, students in the analytical sample nominated 2.25 classmates, and 24.96% nominated the full five. Based on the rejection nominations, directed rejection networks were constructed for all classrooms and used to calculate a rejection score for each individual student. The rejection score represents the number of rejection nominations each student received, divided by the number of participating classmates (the highest number of rejection nominations a student can receive). Thus, scores range between values of zero and one.

#### Student characteristics

##### Migration background

Students with at least one Swedish-born parent are classified as of Swedish origin (1), while students with both parents born outside of Sweden are classified as of immigrant background (0), consistent with the official definition used by Statistics Sweden and prior research in the Swedish setting (e.g., Plenty & Jonsson, 2017).^3^ Information was drawn from student reports on parental country of origin. Missing data in the first wave was replaced by information in the next available wave of the CILS4EU. The last available report was used in case of inconsistent reports across waves.

##### Gender

Gender separates between males (0) and females (1). Information on gender came from survey data.

##### Household income

Household income reflected the total post-tax income from labor, capital, and social benefits of custodial parents in the year 2010. Information on household income came from administrative register data. The income measure was top-coded to three standard deviations from the mean and instances of negative and zero income were coded as missing. In cases where parents lived in separate households the measure represents the mean of the two households. The measure is expressed in 100,000’s of SEK.

##### Parental education

Information on parental education came from administrative register data, thus capturing the highest achieved educational degree. The variable was recoded to represent years of education for the parent with the longest education.

##### Cognitive test score

In addition to the sociodemographic characteristics, the study also assess cognitive ability as a predictor, because it likely influences how attractive a student is to sit next to given the scholastic setting of a classroom. Cognitive ability was assessed using the “Culture Fair Intelligence Test”, a timed pattern recognition test (CFT20R; see Weiß, 2006) with possible test scores ranging between 0 and 27.

#### Classroom Characteristics

Classroom-level variables were calculated as the aggregated means of each of the four student sociodemographic characteristics and cognitive ability. These represented the share of Swedish origin students and female students in each class, as well as the class mean household income and mean number of years of education for students’ most highly educated parent, respectively. For cognitive ability, this represented the mean cognitive ability of the class. Table 1 presents the descriptive statistics of our explanatory variables.

**Table 1:** Descriptives and intraclass correlations for rejection and explanatory variables

	Individual level Mean (Std)	Classroom level Mean (Std)	ICC (Std)
Peer rejection score (0 to 1)	0.100 (0.134)	0.100 (0.035)	0.020 (0.006)
Migration background (1 = Swedish origin)	0.669 (0.471)	0.666 (0.290)	0.354 (0.029)
Gender (1 = Girl)	0.507 (0.500)	0.503 (0.116)	0.015 (0.007)
Household income (in SEK 100,000)	4.779 (2.622)	4.750 (1.269)	0.210 (0.022)
Parental education (in years)	12.726 (2.698)	12.682 (1.145)	0.147 (0.015)
Cognitive test score (0 to 27)	17.712 (4.850)	17.697 (2.012)	0.133 (0.018)

Descriptives are unweighted. Rejection score mean is identical at the individual and classroom-level due to the with-in classroom standardization of the score; Individual level *n* = 4215, except for household income (*n* = 4181), parental education (*n* = 4166), and cognitive test score (*n* = 4037). Classroom-level *n* = 4215, for all characteristics; ICCs estimated by mixed command in Stata

### Analytical Strategy

#### Analysis of who receives peer rejection nominations: Multilevel random effects models

The first part of the analysis examines whether the classroom composition of a given characteristic moderates the degree to which a student with that characteristic is rejected (e.g., whether girls receive fewer rejections when the share of girls in the classroom is larger). Multilevel random intercept models are used, with cross-level interactions representing the moderating influence of classroom composition on the individual-level characteristic. For ease of interpretation, household income, parental education, and cognitive ability are grand mean centered. To obtain correct standard errors while including cross-level interactions, robust standard errors are clustered at the classroom-level.

#### Analysis of who rejects whom: ERGM specification and metaregression

The second part of the analysis examines outgroup derogation by specifying a directed Exponential Random Graph Model (ERGM), examining the network structure of rejections at the classroom-level. The ERGM produces a unique set of estimates for each classroom. Following Smith et al. (2016), metaregression is used to estimate average parameters across classroom networks and include classroom-level characteristics as explanatory variables to examine whether the ERGM parameters are moderated by classroom characteristics (see also Snijders and Baerveldt 2003).

ERGMs are statistical models for examining the structure of ties in social networks (Robins et al., 2007). In essence, ERGMs make it possible to examine if students with certain characteristics are more likely than their counterparts to reject and be rejected by classmates, while controlling for characteristics of the network structure that shape rejection independently of student characteristics. The specification of the network structure was informed by theory (Lusher & Robins, 2013; Robins et al., 2009) and previous network studies describing negative peer relationships (e.g., Boda & Neray, 2015; Harrigan & Yap, 2017; Huitsing et al., 2012; Wittek et al., 2020). The specification also reflects an iterative process (on a sub-sample of networks), whereby structural network parameters impeding model convergence were removed and goodness-of-fit statistics were used to inform decisions to include additional structural parameters. As the focus of the study is on parameters relating to the student characteristics, details on the specification of the network structure, as well as model convergence and goodness-of-fit are presented in the Supplementary material (Appendix A). Because students could nominate a maximum of five classmates, this constraint was imposed on the ERGMs.

Rejection tie formation attributable to student characteristics consists of three components: 1) how differences in the probability of rejecting a classmate depends on rejector’s characteristics (sender effect); 2) how difference in the probability of being rejected depends on the rejected’s characteristics (receiver effect); and 3) differential tendency for students with a certain characteristic to reject others with the same characteristics (interaction effect). Significant sender and receiver effects will represent relative difference in rejection activity (sender) and rejection probability (receiver), whereas a significant interaction effect represents differences in rejection behavior conditional on the characteristics of both receiver and sender (hetero-/homophily). For migration background and gender, these interactions effectively represent heterophily (rejecting different-characteristic peers, i.e., outgroup derogation) if the parameters are negative, and homophily (rejecting same-characteristic peers) if the parameters are positive. The interaction effect should be interpreted jointly with the sender and receiver effect. To do so, conditional odds ratios are calculated (Robins & Daraganova, 2013, pp. 96–98). For household income, parental education, and cognitive test scores, absolute difference terms are used, with positive parameters indicating heterophily (that students are increasingly likely to reject someone when the difference between them is larger).

## Results

### Descriptive Results

**Fig. 1:**
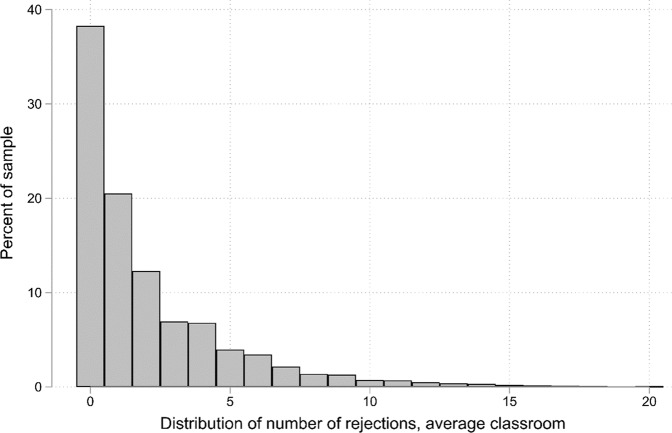
The distribution of rejections in an average classroom of 21 students. Figure shows the peer rejection score multiplied with the average classroom size in the analytical sample (21 students)

Figure 1 shows the distribution of the rejection score, scaled by the average sized classroom in the data (21 students). Almost 40% of students in the average classroom received no rejection nominations, with the share of students decreasing as the number of received rejection nominations increased. Thus, large variation in the number of rejection nominations that students received exists within classrooms. Table 1 (above) presents the share of variance for each of the characteristics that is attributable to between classroom differences by using unconditional intraclass correlations (ICC). For peer rejection, the ICC is close to zero, indicating that almost all variation occurs within classrooms. Nevertheless, the distribution of rejection based on sociodemographic characteristics could still vary between classrooms.

### Results from Multilevel Random Effect Models: Who Receives Peer Rejection Nominations?

Turning to the first part of the analysis on the moderating effects of classroom-level characteristics on the number of rejection nominations a student receives, Table 2 presents the results from the multilevel random intercept model with students’ rejection score as the dependent variable. Model 1 includes only individual-level characteristics, Model 2 in addition includes classroom-level characteristics, and Model 3 adds cross-level interactions. As the rejection score is bounded between zero and one, the estimates can be interpreted as percentage-point changes (divided by 100) in the extent of rejection when a covariate increases with 1. Given that the average classroom size is 21 students, a change in 1 percentage point in the rejection score is the equivalent of a change in 0.2 rejection nominations on average and the results are presented as such.^4^

**Table 2:** Multilevel random intercepts model predicting individual peer rejection score using individual and classroom-level characteristics (*n* = 3982)

	Model 1	Model 2	Model 3
Individual characteristics			
Migration background (Swedish origin)	−0.016** (0.006)	−0.035** (0.006)	−0.036** (0.007)
Gender (Girl)	−0.017** (0.006)	−0.017** (0.006)	−0.018** (0.005)
Household income (in 100 000 SEK)	−0.004** (0.001)	−0.005** (0.001)	−0.005** (0.001)
Parental education (in years)	−0.001 (0.001)	−0.002* (0.001)	−0.002* (0.001)
Cognitive test score	−0.004** (0.000)	−0.004** (0.001)	−0.004** (0.001)
Classroom characteristics			
Share with Swedish origin		0.062** (0.013)	0.083** (0.019)
Share of girls		0.012 (0.022)	0.091** (0.035)
Mean household income		0.004 (0.003)	0.003 (0.003)
Mean years of parental education		0.005 (0.004)	0.005 (0.004)
Mean cognitive test score		0.002 (0.002)	0.001 (0.002)
Cross-level interactions			
Swedish origin*Swedish share			−0.038+ (0.021)
Gender*Share of girls			−0.143** (0.044)
Household income*Mean hh. inc			0.001+ (0.000)
Parental education*mean yrs par. educ.			−0.000 (0.001)
Cognitive test score*Mean cognitive test score			−0.001** (0.000)
Constant	0.119** (0.006)	0.131** (0.006)	0.138** (0.007)

+*p* < 0.10, *p < 0.05, **p < 0.01

Starting with Model 1, students of Swedish origin, girls, and students with higher household income, and with higher cognitive ability all received significantly fewer rejection nominations than their counterparts did, but there is no association with parental education. On average, Swedish origin students were rejected by 0.32 (0.016*20) fewer classmates than students of immigrant background, girls received an average of 0.34 fewer nominations than boys, each 100,000 SEK ( ≈ €10,000, in 2010) increase in household income was associated with receiving 0.08 fewer rejection nominations, and a 1 standard deviation increase in cognitive ability was associated with 0.38 (-.004*4.850*20) fewer rejection nominations.

In Model 2, students in classes with a higher share of Swedish origin students had larger peer rejection scores, keeping individual-level characteristics fixed. Also, the coefficient for Swedish origin more than doubles in size when accounting for classroom-level variables, with Swedish students receiving 0.7 fewer nominations. In other words, when accounting for the fact that Swedish origin students often attend school classes with a higher share of Swedish origin students (classes which also tended to have a higher level of rejection, all else equal), the individual-level association between migration background and peer rejection becomes stronger. Similarly, the individual-level association between parental education and rejection increases somewhat in size when accounting for classroom-level characteristics.

**Fig. 2:**
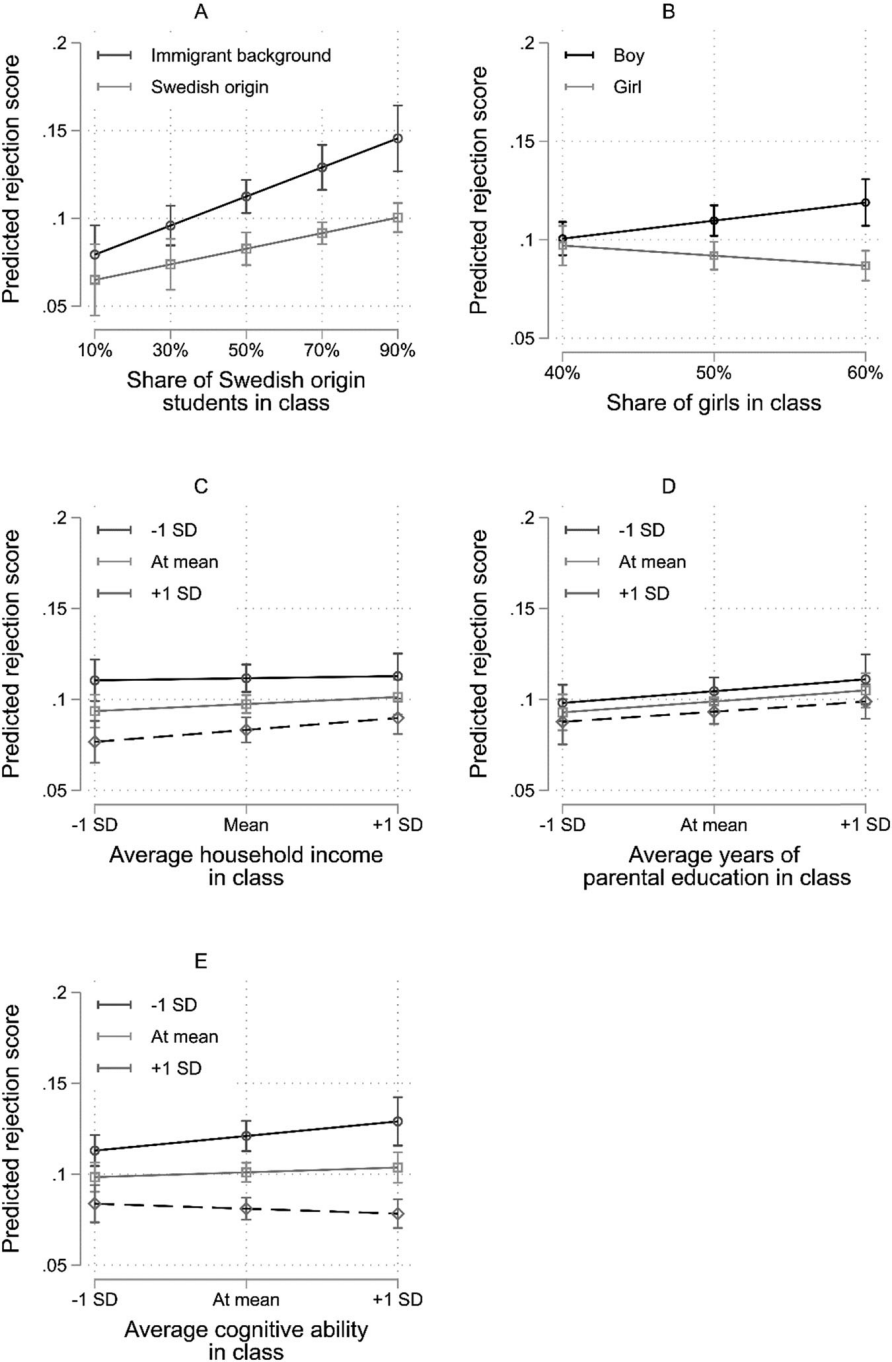
Linear predictions of the rejection score, illustrating the cross-level interaction effects from Table 2, Model 3 (*n* = 3982). All classroom-level variables were mean centered. **A** and **B** has different x-axes to reflect their span of meaningful classroom-level variation in the data. See results from Table 2 for significance of group differences

In Model 3, there are significant interactions between the individual- and classroom-level characteristics for migration background, gender, household income, and cognitive ability (migration background and income are significant at the 10% level; *p* = 0.062 and 0.072, respectively). Figure 2 illustrates the interaction effects from Model 3. Both students of immigrant background and students of Swedish origin received more rejection nominations in classrooms with a higher share of Swedish origin students, but as the share increased, the increase in received rejection nominations was stronger for immigrant background students than Swedish origin students. In a classroom with 10% Swedish origin students, immigrant background students receive 0.3 more rejections than their Swedish origin counterparts, but in a classroom with 90% Swedish origin students, immigrant background students receive 0.90 more rejections. In classrooms with a lower share of girls (and thus higher share of boys, 60%), girls and boys received a similar number of nominations. But in classrooms with a higher share of girls (60%), boys received 0.6 more rejection nominations than girls. Household income followed a different pattern, as indicated by the positive cross-level interaction coefficient. Higher income was slightly more protective in classes with lower average income than in classes with higher income. Also, students with lower cognitive ability received more rejection nominations in classrooms with higher average cognitive ability than in classrooms with lower average cognitive ability.

Thus, Hypothesis 1 was partially supported, as immigrant background students, boys and girls, and students with lower cognitive ability received more rejection nominations in school-classes where their respective characteristic was less common. Contrary to expectations, Swedish origin students received fewer rejection nominations in absolute terms in classes with a lower share of Swedish origin students, but this was due to a lower overall amount of rejection in the classrooms instead of Swedish students receiving a lower share.

### Results from ERGMs and Metaregression: Who Rejects Whom?

Turning from predicting students’ peer rejection scores to examine the social dynamics shaping rejection ties between certain students, the specified model converged for 175 out of the 201 classroom networks, and an additional 20 networks were excluded due to non-satisfactory goodness-of-fit statistics (for details, see Supplementary material, Appendix A). In all, 155 networks were included in the metaregression analyses. To ensure internal consistency, the Multilevel Random Effects models were re-estimated using only the 155 classrooms included in the ERGM analysis. Results remain the same, as can be seen from Table B1 in the Supplementary material.

Although the ERGM specification was intended to be consistent across classroom networks, some configurations did not exist within all networks. For example, classes without any immigrant background students could not have migration background-based sender, receiver or heterophily parameters. When a configuration did not exist, it was excluded from the specification for that classroom network, which lead to differences in the number of classroom networks estimated for each parameter in the metaregression.

Table 3 presents results from the metaregression on the ERGM estimates. Hypothesis 2 states that students would send more rejection nominations to different-characteristic classmates than to same-characteristic classmates. From Table 3, migration background had significant positive sender and significant negative interaction parameters, while gender had significant sender (positive), receiver (positive), and interaction (negative) parameters. These results are discussed in more detail below.

**Table 3:** ERGM estimates summarized across classrooms obtained using metaregression

Parameter	Estimate	SE	*N*	*I*^2^
Migration background				
Swedish origin Sender	1.016**	0.331	134	0.869
Swedish origin Receiver	0.463	0.310	134	0.873
Sender*Receiver	−0.848*	0.365	121	0.901
Gender				
Girl Sender	0.616**	0.133	155	0.724
Girl Receiver	0.521**	0.168	153	0.780
Sender*Receiver	−1.509**	0.195	133	0.785
Household income				
Income Sender	−0.001	0.013	155	0.305
Income Receiver	−0.033*	0.014	155	0.523
Absolute difference	0.004	0.012	155	0.230
Parental education				
Par. educ. Sender	0.003	0.009	155	0.226
Par. educ. Receiver	−0.012	0.009	155	0.349
Absolute difference	−0.007	0.008	155	0.046
Cognitive test score				
Cog. test Sender	0.027**	0.006	155	0.425
Cog. test Receiver	−0.022**	0.005	155	0.418
Absolute difference	−0.007	0.005	155	0.305

A corresponding table including also network structure estimates are available in the Supplementary material (Table A2)

+*p* < 0.10, *p < 0.05, **p < 0.01

Household income had a significant negative receiver parameter but not a significant sender nor absolute difference parameter. A 100,000 SEK increase in household income was associated with 0.033 lower odds for receiving a rejection nomination. Thus, students from homes with lower household income were more likely to be the target of rejections but income did not relate to the tendency to send rejections nor is there any indication that students were more likely to reject classmates more dissimilar to themselves on income (as captured by the absolute difference parameter). Higher cognitive ability was associated with a greater tendency to reject others (0.027 higher odds per increase in 1 on the test), and a lower tendency to be rejected by others (0.022 odds per increase in 1 on the test), but no evidence of a joint effect from the sender and the receiver’s cognitive ability. No estimates are significant for parental education.

To facilitate the interpretation for migration background and gender, Table 4 reports calculated odds ratios using the sender, receiver, and interaction parameters. Consistent with Hypothesis 2, there is a significant tendency for outgroup derogation by Swedish origin students: Swedish origin students rejected immigrant background classmates 0.9-fold more than they rejected Swedish origin classmates. Despite a trend for students of immigrant background to reject Swedish origin classmates more than immigrant background classmates, this difference was not statistically significant at traditional significance levels. Only Swedish origin-to-immigrant background significantly differs from the baseline (immigrant-to-immigrant), indicating that a student of immigrant background was substantially more likely to be rejected by a student of Swedish origin than immigrant background, whereas a Swedish origin student was equally likely to be rejected by a Swedish origin or immigrant background student. For migration background, there is partial support for Hypothesis 2.

**Table 4:** Odds ratios for rejection nominations

Sender	Receiver		Outgroup derogation: Wald tests	
Migration background	Immigrant (0)	Swedish (1)		
Immigrant (0)	1	1.589	imm-imm vs. imm-Swed	n.s.
Swedish (1)	2.762	1.879	Swed-imm vs. Swed-Swed	*p* < 0.05
Gender	Boy (0)	Girl (1)		
Boy (0)	1	1.701	boy-boy vs. boy-girl	*p* < 0.05
Girl (1)	1.684	0.633	girl-boy vs. girl-girl	*p* < 0.05

Gender showed a significantly higher tendency for outgroup derogation: boys reject girls more than they reject boys and girls reject boys more than they reject girls (0.7-fold higher odds for both genders). Further, although both genders demonstrate outgroup derogation, it was stronger among girls than among boys, as indicated by the findings that girls rejecting boys was as likely as boys rejecting girls, but girls rejecting girls was 0.7-fold less likely than boys rejecting boys (all gender-combinations are significantly different from the baseline of boy rejecting boy at a 5% level).

Hypothesis 3 posited that the tendency to reject different-characteristic classmates would be stronger in classes where the prevalence of the dissimilar characteristic is lower. Table 5 shows significant estimates for migration background, gender, and cognitive test score. The results in Table 5 describe variation in the average parameter estimates reported in Tables 3 and 4 and should therefore be interpreted jointly with these tables. For migration background, Tables 3 and 4 reported that on average Swedish origin students rejected classmates with an immigration background more, but not vice versa. Table 5 shows that as the share of Swedish origin students in a classroom increased (i.e., where immigrant background students were less prevalent), the likelihood of Swedish origin students sending rejection nominations increased, whereas the interaction decreased. Swedish origin students increasingly rejected immigrant background classmates as the share of Swedish origin students increased. To a lesser and nonsignificant extent, immigrant background students’ outgroup derogation also increased in classes with a higher share of Swedish origin students. The large (in absolute sense) and significant estimate for the interaction among Swedish origin students generally lead to Swedish origin students’ outgroup derogation increasing as their share increased in a classroom. Thus, there is partial support for Hypothesis 3 according to migration background.

**Table 5:** ERGM estimates summarized across classrooms obtained using metaregression with classroom-level characteristics as covariates

Parameters	Estimate	Classroom characteristic	*N*
Migration background			
Swedish origin Sender	3.059^*^ (1.354)	Share of Swedish origin	134
Swedish origin Receiver	1.373 (1.260)		134
Sender*Receiver	−3.750^*^ (1.840)		121
Gender			
Girl Sender	3.492^**^ (1.166)	Share of girls	155
Girl Receiver	3.382^*^ (1.466)		153
Sender*Receiver	−2.173 (1.782)		133
Household income			
Income Sender	−0.006 (0.010)	Income (mean)	155
Income Receiver	0.005 (0.010)		155
Absolute difference	−0.006 (0.008)		155
Parental education			
Par. educ. Sender	−0.001 (0.008)	Parental education (mean)	155
Par. educ. Receiver	−0.008 (0.008)		155
Absolute difference	0.003 (0.007)		155
Cognitive test score			
Cognitive test Sender	0.001 (0.003)	Cognitive test (mean)	155
Cognitive test Receiver	−0.006^*^ (0.003)		155
Absolute difference	−0.004 (0.003)		155

All classroom-level variables were mean centered

+*p* < 0.10, *p < 0.05, **p < 0.01

When the share of girls in the classroom was higher, so was the relative estimates of rejection of girls (receiver effect) and by girls (sender effect), relative to boys rejecting boys. The smaller (in absolute sense) and nonsignificant interaction estimate further shows that girls’ tendency to reject girls ‘increased’ faster than their tendency to reject boys as the classroom composition skewed more towards girls. That is, girls’ tendency to reject boys was smaller in classrooms with a higher share of girls, and boys’ tendency to reject girls was larger, which is in discordance with Hypothesis 3.

An additional and exploratory finding, described in Table A3 in the Supplementary material, is that several network structure parameters vary systematically with the share of girls in the classroom. In classrooms with more girls, it was more common that students sent rejection(s) without themselves receiving any rejection nominations and more common that students neither sent nor received any rejection nominations. Further, there was less variation in the number of rejection nominations students received, likely indicating that rejections were less concentrated to certain individual students. Thus, while there is no evidence for gender compositional effects increasing outgroup derogation, the underlying structure of rejections in the classroom varies with gender composition.

As the average cognitive ability in the classroom increased, students with higher scores were less likely to be rejected by classmates (significant and negative estimate of receiver effect). Given that Table 3 showed a positive sender estimate for cognitive ability and a negative receiver estimate, this shows that as ability increased across classrooms, high ability students remained equally likely to reject peers but were less likely to be rejected by peers, thereby concentrating rejections among less able classmates, but without indications of outgroup derogation. There is no evidence of heterogenous rejection patterns for parental level of education and household income across the classroom mean of these characteristics.

## Discussion

Peer rejection has substantial negative consequences for students’ school engagement (Juvonen et al., 2019; Wentzel et al., 2021) and mental well-being (Timeo et al., 2019). Rejection does not occur randomly, with prior research documenting a higher risk of rejection for students with characteristics such as immigration background (Plenty & Jonsson, 2017); low economic resources (Hjalmarsson, 2018) and low parental education (Knaappila et al., 2018; Nordhagen et al., 2005). This study aimed to advance knowledge on social inequalities in peer rejection by assessing the applicability of person-group dissimilarity and social identity theory processes across a range of sociodemographic characteristics: migration background, gender, household income, and parental education, as well as for cognitive ability.

The first part of the analyses assessed the applicability of person-group dissimilarity theory to explain social inequalities in peer rejection. In line with Hypothesis 1, classroom composition moderated how migration background and gender associated with rejection: Immigrant background students received more rejection nominations in classrooms with fewer immigrant background students and both girls and boys received a larger share of rejection nominations in classrooms where their respective gender was less represented. Thus, the results confirmed that the person-context interactions drawn from person-group dissimilarity theory (Wright et al., 1986) apply to these characteristics. For immigrant background students, the finding is consistent with reports that such students experience more victimization (Plenty & Jonsson. 2017) and greater loneliness (Madsen et al. 2016) in immigrant sparse school settings. For gender-based rejection the study is, to the best of our knowledge, the first to demonstrate the compositional effects predicted by person-group dissimilarity theory. Although the increased rejection of Swedish-origin youth in classrooms with a higher share of Swedish origin youth was unexpected, a previous study found that both Swedish origin and immigrant background students reported worse peer relationships in schools with a lower share of immigrant background students (Hjern et al. 2013). It is not possible to explore the reasons underlying this phenomenon within the current study, but future studies should examine the potential for classrooms with greater ethnic diversity to embody a generally welcoming or cohesive social climate (Juvonen et al. 2006).

Despite suggestions that socioeconomically disadvantaged students may experience socioemotional difficulties in school contexts with few same-characteristic peers (Benner & Wang, 2014; Crosnoe, 2009), students with low household income and students with lower educational background received a similar number of rejection nominations across classroom settings. If anything, household income was slightly more protective against rejection in low-income settings than in high-income settings.

The second part of the analyses assessed processes drawn from social identity theory, by examining the role of outgroup derogation for social inequalities in peer rejection. The theoretical expectation was that students would send more rejection nominations to different-characteristic classmates than same-characteristic classmates (Hypothesis 2). Consistent with Hypothesis 2, the study found evidence of outgroup derogation based on migration background (by students of Swedish origin) and on gender (by boys and girls), but no statistically significant evidence of outgroup derogation by students of immigrant background; nor any indication of outgroup derogation based on household income, parental education, or cognitive ability. Thus, in terms of social identity theory (Tajfel, 1982; Tajfel & Turner, 1979), social categories relating to whether one belongs to the majority population and gender are associated with the tendency to distance oneself from “outgroup” members. However, it is likely that the motivations underlying outgroup derogation according to these characteristics differs, as outgroup derogation of immigrant background students is in line with the literature on prejudices towards migrants (e.g., Miklikowska, 2016), while for gender, a more subtle (and, perhaps fully age-expected) type of avoidance behavior may take place (Leaper, 2013). Importantly, rejection does not reflect a simple preference to favor same-characteristic classmates, but instead represents a willingness to avoid different-characteristic classmates, which provides a challenge for building positive intergroup relationships. Yet it remains unclear whether this outgroup derogation is intentional, implicit or overlaps with other attributes such as behaviors or interests. Nevertheless, schools’ efforts to promote the social integration of immigrant background students should address exclusionary behaviors by students of Swedish origin and efforts to promote positive gender relations could benefit by addressing these outgroup biases in boys’ and girls’ interpersonal preferences (cf. Juvonen et al., 2019).

The analysis continued by assessing the role of outgroup derogation in producing the compositional effects predicted by person-group dissimilarity theory and observed in the multilevel models. Hypothesis 3 stated that students’ outgroup derogation would be stronger in classes with a lower share of different-characteristic classmates. In relation to migration background, this hypothesis received partial support. Swedish origin students’ outgroup derogation was stronger in classrooms with a lower share of immigrant background students. On the other hand, immigrant background students were not more likely to nominate students of Swedish origin in classrooms with a smaller share of Swedish origin students. For gender, the study found the opposite relation as expected from Hypothesis 3—the tendency for outgroup derogation by boys and girls was weaker in classrooms where the opposite gender was less represented. No evidence for the classroom composition moderating outgroup derogation for income, parental education, or cognitive ability was found.

Bringing the results together, both person-group dissimilarity and social identity theory seem applicable to migration background and gender-based rejection, person-group dissimilarity was applicable to cognitive ability, but neither applied to socioeconomic-based rejection. Thus, school policy and interventions aiming to improve ethnic inter-group relations should address potential discrimination from students of majority background, particularly in school contexts with fewer immigrant background students. However, other explanations than increasing gender-based outgroup derogation are needed to understand the higher rejection of boys or girls in such classes—something buttressed by the finding that the broader structure of rejection networks varied somewhat across classrooms of varying gender composition. Future research could further examine how the gender composition of a classroom is associated with the social dynamics driving rejection between students, especially adolescent students, for which their emerging self-identities have large gender components.

Person-group dissimilarity and social identity processes appear less important for socioeconomic-based rejection, as this was mostly constant across settings and rejections were not more likely between different-characteristic classmates. Compared to categories of migration background and gender, household income and parental education are less visibly discernible and likely also less central for self-identity formation. The generally higher rejection of students from lower income households might instead arise due to wealth being perceived as high status and desirable among individuals from across the income distribution. This is consistent with studies showing tendencies for children to favor affluent peers, regardless of their own socioeconomic background or school-class composition (la Roi et al., 2023; Shutts et al., 2016). Accordingly, the protective effects of higher income in low-income classroom settings may arise if the prestige of higher income becomes amplified in contexts where it is less common. This identifies a need for school policy to develop strategies and social activities that can improve the social inclusion of economically disadvantaged students, regardless of the school’s economic composition (Juvonen et al., 2019). Furthermore, although some studies have found that adverse peer relationships are related to low parental education (e.g. Nordhagen et al., 2005), by simultaneously testing different indicators of socioeconomic background, this study found that household income was the predictive socioeconomic factor of rejection. If visibility is indeed an important component, this may be partly due to income having more tangible indicators (e.g., brand clothes, activities, consumption) compared to parental education (e.g., cultural resources).

Using a large, high-quality dataset the current study found students with an immigration background, boys, and students of lower household income to be rejected to a larger extent than other students. Drawing on social identity theory and person-group dissimilarity theory the current study extended prior research by assessing person-context interactions in the receipt of peer rejection for multiple sociodemographic characteristics while also examining the social dynamics underlying these patterns. In doing so, it provided a more comprehensive picture of social inequalities in peer rejection than has previously been presented. It also responded to recent calls for incorporating person-context interactions and sociodemographic characteristics to better understand peer relationships (Bukowski et al., 2021).

Yet, neither the data nor the empirical strategies are without important limitations. First and foremost, the study focused on 14–15 years old students living in Sweden. The age of the respondents may affect findings: Particularly for gender-based outgroup derogation, differences in adolescent development could account for the difference in patterns across gender. Future studies could aim to replicate this study with younger and older participants to clarify the extent to which gender-based outgroup derogation weakens or persists across the school years. Similarly, replication of findings in countries with different gender norms, immigration policies, and level of economic inequality could elucidate whether the patterns differ across country contexts. Second, while the simultaneous analysis of multiple sociodemographic characteristics was a key strength of the study, previous research has identified student behaviors and attitudes (e.g., aggression, prosocial behavior, prejudices) that are often associated with adverse peer relationships. Future research should examine the role of these mechanisms as potential mediators linking sociodemographic characteristics to rejection.

Third, the operationalization of migration background was crude but necessary for statistical efficiency. Results could potentially vary according to generation (first or second), the timing of arrival, as well as region of origin, language, and cultural background. It should, for instance, be kept in mind that in classes with a low representation of Swedish origin students there is typically a greater ethnic diversity, so Swedish origin students do not necessarily constitute a numerical minority in terms of ethnicity in such classes (Plenty & Jonsson, 2017). Other studies have better addressed the experience of specific ethnic groups (e.g., Boda & Neray, 2015) and future studies taking such an approach should be better positioned to also assess the importance of issues such as generalized prejudice (Allport, 1954) versus marginalized-group prejudice (Bergh et al., 2016) and group-specific prejudice (Duckitt & Sibley, 2007), which unfortunately was beyond the scope of the current study. Future studies could further unpack which immigrant background characteristics (e.g., language use) or subpopulations (e.g., first- or second generation) are more or less sensitive to person-group dissimilarity dynamics.

Fourth, peer rejection was based on a sociometric question asking students to report classmates whom they did not want to sit next to. This measure has the benefit of capturing a broad range of rejections, from the subtle to the more explicit. However, while even subtle experiences of rejection produce negative emotions (Timeo et al., 2019), more explicit forms of rejection are likely to more strongly affect mental well-being and school-engagement and may be more salient to address in terms of policies. Future research should assess whether the patterns are replicated when using measures capturing less subtle and likely more explicit rejection, such as dislike or victimization nominations. Similarly, while most students (75%) utilized less than the maximum of five rejection nominations, future research could allow an unrestricted number of nominations to assess whether this affects results. Last, the study was unable to address the possibility of intersectionality in peer rejection, partly due to methodological concerns, and partly due to the scope of the present paper. Yet, future studies of peer rejection should be attentive to whether there exist specific rejection dynamics playing out in especially the class-immigrant-gender nexus.

## Conclusion

While sociodemographic characteristics are associated with a greater risk of peer rejection at school, it is unclear how key theoretical frameworks explaining rejection apply across a range of characteristics. This study found that migration background, gender and cognitive ability related to person-group dissimilarity processes. The rejection of immigrant background students as well as boys and girls also related to social identity theory processes. The combination of the two theoretical models revealed that Swedish origin students’ stronger outgroup derogation in immigrant sparse school-classes could underly the higher rejection of immigrant background students in such settings. Thus, adolescents’ adverse peer experiences stem from a range of characteristics, some of which are entwined with intergroup relations and person-context interactions. Educators should be mindful of social inequalities in students’ peer relationships, and how vulnerability may differ across school settings. A policy priority should be to address biases held by majority background students, particularly in settings with a lower representation of immigrant background students.

## Supplementary information

Supplementary Materials

## Footnotes

1 Data from the second and third CILS4EU wave were used to replace missing data on migration background.

2 Self-nominations and instances where students reported the same classmate several times were removed. Nominations to classmates who the student also nominated as a friend were also removed (163 students made 296 such nominations, representing 3% of total nominations).

3 Of students in the analytical sample with an immigration background, 64% were born in Sweden while 36% were born outside Sweden. Reflecting the heterogenous Swedish immigrant background population (Jonsson, 2018), parental region of origin was diverse, with 5% of immigrant background students originating from Northern Europe, 18% from Southern Europe; 7% from Eastern Europe; 4% from South America; 9% from Asia; 40% from the Middle East; 13% from Africa, and 4% from other regions.

4 Students cannot reject themselves, so the maximum possible number of rejection are 20.
