# Supplementary material for: Not Next to You: Peer Rejection, Sociodemographic Characteristics and the Moderating Effects of Classroom Composition
Source: J Youth Adolesc. 2023 Mar 10;52(6):1191–205. doi: 10.1007/s10964-023-01758-x (PMC10121533; doi:10.1007/s10964-023-01758-x)
Supplement: Supplementary file 1 — Supplementary Materials [file 10964_2023_1758_MOESM1_ESM.docx]

**Supplementary material**

**Not Next to You: Peer Rejection, Sociodemographic Characteristics and the Moderating Effects of Classroom Composition**

# Appendix A: The Exponential Random Graph Models

## ERGMs and the ERGM specification

Exponential Random Graph models (ERGMs) are a type of regression models used for analyzing network data consisting of links (relationships) between nodes (in our case, students). The unit of analyses are not the nodes, but instead the probability of a link between nodes. In an ERGM framework, networks are composed of local patterns of network ties (termed configurations), corresponding to assumptions about how the propensity for certain ties depend on the presence of other ties as well as on the characteristics of actors in the network. An ERGM is specified through the inclusion of configurations, and parameters are estimated by applying pseudo maximum likelihood models to an observed network.

To accurately make inferences about the role of student characteristics in shaping peer rejection in the classroom, we control for characteristics of the network structure that reflect social rules governing rejection independently of student characteristics. Our ERGM specification thus includes student (actor) characteristics, between-student characteristics, as well as network structure characteristics. Because each student can nominate a maximum of five classmates, we restrict the ERGM to a maximum of five outgoing rejection nominations per student. We restrict the maximum number received rejections for each student to n-1, where n is the number of students in the classroom.

Table A1 presents an overview of the configurations included in our ERGM specification. As student and between-student characteristics are described in the paper we here focus on the specification of the network structure. We developed our ERGM specification of the network structure based on theory of the structural characteristics of negative peer relationships (Lusher & Robins, 2013; Robins, Pattison and Wang, 2009) as well as the specifications used in prior network studies of negative peer networks (e.g., Boda & Neray, 2015; Harrigan & Yap, 2017; Huitsing et al., 2012; Wittek et al., 2020). In addition, we developed our specification through an iterative process (using a sub-sample of networks), removing structural network parameters impeding model convergence and making decisions on including additional structural parameters on the basis of goodness-of-fit statistics.

**Table A.1** Configurations modelled in ERGM

| *Actor attributes* | *Network structure attributes* |
| --- | --- |
| *Migration background* | Arc |
| Swedish origin Sender | Reciprocity |
| Swedish origin Receiver | 2-path |
| Sender*Receiver | 2-in-star |
|  | Sink |
| *Gender* | Source |
| Girl Sender | Isolates |
| Girl Receiver | AinS(2.00) |
| Sender*Receiver | AoutS(2.00) |
|  | AT-D(2.00) |
| *Household income* |  |
| Income Sender |  |
| Income Receiver |  |
| Absolute difference |  |
|  |  |
| *Parental education* |  |
| Par. educ. Sender |  |
| Par. educ. Receiver |  |
| Absolute difference |  |
|  |  |
| *Cognitive test score* |  |
| Cog. test Sender |  |
| Cog. test Receiver |  |
| Absolute difference |  |
|  |  |

As the first structural parameter, we include a constant term capturing the propensity of rejection nominations in the network (arc), which is needed to examine if the baseline likelihood of the formation of rejection ties varies across classrooms. Second, we model reciprocity, which captures if students are more likely than at random to mutually nominate each other. Reciprocity is a strong driver of positive ties (such as friendships), but whether this is the case also for negative ties (such as rejection) is more contested (see review in Harrigan & Yap, 2017). We also include parameters that capture circuits connecting three students (i.e., 2-paths) and the case where two students nominate the same classmate (i.e., in-2-star). On the other hand, we do not include network statistics capturing transitivity (mechanisms of the type “a friend of a friend is more likely to be a friend”). Transitivity is an important driver of positive ties, but previous research has not found evidence of transitivity being a significant driver in the formation of negative ties (e.g., Harrigan & Yap, 2017; Lusher & Robins, 2013).

We also include a parameter to capture students who neither receive nor send rejection nominations (isolates), since negative networks tend to be much more sparse than positive networks. We also include a parameter capturing students who only receive but never send rejection nominations (sinks), and students who reject others but never receive rejections nominations (sources). Further, incoming rejection nominations are likely concentrated among some students who are more unpopular than others, so we also model this “inequality-in-nominations”-effect for our classroom networks (alternating in-star). Similarly, as some students may reject more peers than others do, leading to a skewed distribution in outgoing nominations, we include a parameter to capture this spread in rejection nominations (alternating out-star). Finally, we found that more of our networks converged and model fit was improved when we included a structural homophily parameter that captures shared unpopularity (i.e., AT-D). We can think of the shared unpopularity parameter as capturing students’ propensity to reject the same set of students that their other classroom peers reject (Robins, Pattison and Wang, 2009). For the alternating in-star (A-in-S), alternating out-star (A-out-S), and the shared unpopularity (AT-D) parameters we set the decay parameter (lambda) to equal 2.

## Estimation, convergence, and goodness-of-fit

ERGMs were estimated using the PNet software (Wang et al., 2009). Our specified model converged for 175 out of the 201 classroom networks. In these networks, the convergence t-ratio was below 0.1 for all included parameters (Robins & Lusher, 2013). Based on each fitted model, we simulate a distribution of networks and assessed goodness-of-fit by comparing the observed classroom network with characteristics of the distribution of networks predicted by our model. At this stage, t-ratios for parameters included in our model should ideally not exceed 0.1, but some deviation is acceptable (Robins & Lusher, 2013, p. 181). Initially, 135 networks had included parameter estimates with t-ratios above 0.1 (76 of which also had t-ratios exceeding 0.2). Following Robins & Lusher (2013) we ran a larger goodness-of-fit estimation (using 5 000 000 simulations per network taking a sample of 1 000 networks) on these 135 networks. In the end, we exclude 18 networks where any parameter included in our model has a t-ratio of more than 0.2 (36 retained networks have t-ratios above 0.1 but not exceeding 0.2).

We also assess goodness-of-fit against parameters not included in our model. Here the t-ratio should ideally not exceed 2, but again, some deviation is acceptable. We assessed goodness-of-fit against statistics of graph counts (and associated statistics), degree distributions, clustering, and counts of triads, following recommendations for directed networks in Robins, Pattison and Wang (2009) and Robins and Lusher (Robins & Lusher, 2013). We exclude 2 classroom networks with at least one parameter t-ratio above 3 (16 retained networks have t-ratios exceeding 2 but not exceeding 3). In all, 155 networks were included in the metaregression analyses.

Network models place high demand on the data used and having to discard 46 classrooms networks out of 201 is a substantial sample restriction. However, compared to other similar studies, we retain substantially higher number of networks and sample size (e.g., Boda & Neray, 2015). As noted in the paper, results from the Multilevel Random Effects models remain the same when the sample is restricted to the 155 classrooms included in the ERGM analysis (as can be seen from Table B1 in the Supplementary material).

## Full ERGM Metaregression Results

Table A2 present full results for the meta-regression of our ERGMs. As results for student and between-student characteristics are described in detail in the paper, we here focus on the network structure variables. We find that two students mutually rejecting each other (reciprocity) was significantly more common than would be expected at random. We also see that two students rejecting the same other classmate was more common than expected at random (positive parameter for 2-in-star). There was more dispersion in the number of received rejection nominations than expected at random (positive parameter for A-in-S), likely reflecting the concentration of rejection nominations sent to certain individual students. However, situations with multiple overlaps in which classmates chose to reject sets of students occurred to a lesser extent than expected at random (negative parameter for AT-D), so although students often rejected the same individual students, there is no indication that students ganged up on groups of peers (after controlling for individual student characteristics). Further, there was somewhat more variation than expected in the number of rejection nominations students sent (positive parameter for A-out-S). Last, compared to at random, it was more likely that students received rejections without rejecting anyone themselves (sink), and less likely that they solely rejected others without receiving any rejections (source). The last two results are likely at least partly due to the fact that it was possible to nominate non-present classmates, whom in turn could not nominate anyone themselves, and should likely be considered an artifact of the sampling.

**Table A.2** ERGM estimates summarized across classrooms obtained using metaregression

| Parameter | Estimate | SE | N | I^2^ |
| --- | --- | --- | --- | --- |
| Migration background |  |  |  |  |
| Swedish origin Sender | 1.016** | 0.331 | 134 | 0.869 |
| Swedish origin Receiver | 0.463 | 0.310 | 134 | 0.873 |
| Sender*Receiver | -0.848* | 0.365 | 121 | 0.901 |
| Gender |  |  |  |  |
| Girl Sender | 0.616** | 0.133 | 155 | 0.724 |
| Girl Receiver | 0.521** | 0.168 | 153 | 0.780 |
| Sender*Receiver | -1.509** | 0.195 | 133 | 0.785 |
| Household income |  |  |  |  |
| Income Sender | -0.001 | 0.013 | 155 | 0.305 |
| Income Receiver | -0.033* | 0.014 | 155 | 0.523 |
| Absolute difference | 0.004 | 0.012 | 155 | 0.230 |
| Parental education |  |  |  |  |
| Par. educ. Sender | 0.003 | 0.009 | 155 | 0.226 |
| Par. educ. Receiver | -0.012 | 0.009 | 155 | 0.349 |
| Absolute difference | -0.007 | 0.008 | 155 | 0.046 |
| Cognitive test score |  |  |  |  |
| Cog. test Sender | 0.027** | 0.006 | 155 | 0.425 |
| Cog. test Receiver | -0.022** | 0.005 | 155 | 0.418 |
| Absolute difference | -0.007 | 0.005 | 155 | 0.305 |
|  |  |  |  |  |
| Network structure |  |  |  |  |
| Arc | -4.995** | 0.667 | 155 | 0.943 |
| Reciprocity | 0.724** | 0.077 | 130 | 0.000 |
| 2-path | 0.001 | 0.012 | 155 | 0.261 |
| 2-in-star | 0.156** | 0.022 | 155 | 0.790 |
| Sink | 1.211** | 0.257 | 154 | 0.594 |
| Source | -1.571** | 0.354 | 155 | 0.839 |
| Isolates | -0.140 | 0.454 | 132 | 0.869 |
| AinS | 2.692** | 0.474 | 155 | 0.951 |
| AoutS | 0.396* | 0.152 | 155 | 0.637 |
| AT-D | -0.388** | 0.042 | 151 | 0.364 |

Note: The Table corresponds to Table 3 in the paper but includes results for the network structure configurations. AinS, AoutS, and AT-D all have decay parameter lambda set at 2.0.
 + p< 0.10, * p<0.05, ** p<0.01

| **Table A.3** Variation in Estimates of Network Structure across Classroom Level Characteristics | | | | | | |  |
| --- | --- | --- | --- | --- | --- | --- | --- |
|  | Classroom characteristics | | | | | |  |
| Parameters | Share  of girls | Share of Swedish origin | Income  (mean) | Parental education (mean) | Cognitive test  (mean) | N | |
| Arc | 1.438 (5.792) | 2.848 (2.352) | -0.038 (0.554) | 0.638 (0.587) | 0.339 (0.323) | 155 | |
| Reciprocity | 0.844 (0.644) | -0.352 (0.294) | -0.068 (0.072) | -0.119 (0.073) | -0.032 (0.040) | 130 | |
| 2-path | -0.016 (0.105) | -0.009 (0.052) | 0.001 (0.011) | -0.004 (0.012) | -0.002 (0.007) | 155 | |
| 2-in-star | 0.037 (0.205) | 0.095 (0.113) | 0.007 (0.024) | 0.011 (0.028) | -0.000 (0.014) | 155 | |
| Sink | 2.159 (2.249) | -0.010 (0.919) | 0.224 (0.214) | 0.350 (0.226) | 0.280^*^ (0.123) | 154 | |
| Source | 5.613^+^ (3.058) | 2.880^*^ (1.240) | 0.163 (0.296) | 0.389 (0.314) | 0.033 (0.173) | 155 | |
| Isolates | 10.096^**^ (3.803) | 3.075^+^ (1.556) | 0.434 (0.385) | 0.938^*^ (0.387) | 0.318 (0.216) | 132 | |
| AinS(2.00) | -8.332^*^ (4.072) | -4.015^*^ (1.655) | -0.418 (0.393) | -0.732^+^ (0.417) | -0.294 (0.230) | 155 | |
| AoutS(2.00) | -1.580 (1.338) | -0.363 (0.547) | -0.208 (0.126) | -0.326^*^ (0.132) | -0.164^*^ (0.073) | 155 | |
| AT-D(2.00) | 0.259 (0.359) | 0.041 (0.164) | 0.008 (0.037) | -0.012 (0.039) | -0.012 (0.022) | 151 | |

Note: + p< 0.10, * p<0.05, ** p<0.01

## **References, Appendix A**

Boda, Z., & Neray, B. (2015). Inter-ethnic friendship and negative ties in secondary school. *Social Networks*, *43*, 57–72. https://doi.org/10.1016/j.socnet.2015.03.004

Harrigan, N., & Yap, J. (2017). Avoidance in negative ties: Inhibiting closure, reciprocity, and homophily. *Social Networks*, *48*, 126–141. https://doi.org/10.1016/j.socnet.2016.07.003

Huitsing, G., van Duijn, M. A. J., Snijders, T. A. B., Wang, P., Sainio, M., Salmivalli, C., & Veenstra, R. (2012). Univariate and multivariate models of positive and negative networks: Liking, disliking, and bully–victim relationships. *Social Networks*, *34*(4), 645–657. https://doi.org/10.1016/j.socnet.2012.08.001

Lusher, D., & Robins, G. (2013). Personal Attitudes, Perceived Attitudes, and Social Structures: A Social Selection Model. In D. Lusher, J. Koskinen, & G. Robins (Eds.), *Exponential random graph models for social networks: Theory, methods, and applications* (pp. 189–201). Cambridge University Press.

Robins, G., & Lusher, D. (2013). Illustrations: Simulation, Estimation, and Goodness of Fit. In D. Lusher, J. Koskinen, & G. Robins (Eds.), *Exponential random graph models for social networks: Theory, methods, and applications* (pp. 167–186). Cambridge University Press.

Robins, G., Pattison, P., & Wang, P. (2009). Closure, connectivity and degree distributions: Exponential random graph (p*) models for directed social networks. *Social Networks*, *31*(2), 105–117. https://doi.org/10.1016/j.socnet.2008.10.006

Wang, P., Robins, G., Pattison, P., & Koskinen, J. (2009). *PNet: program for the simulation and estimation of exponential random graph models* (1.0) [Computer software]. Melbourne School of Psychological Sciences, The University of Melbourne.

Wittek, M., Kroneberg, C., & Lämmermann, K. (2020). Who is fighting with whom? How ethnic origin shapes friendship, dislike, and physical violence relations in German secondary schools. *Social Networks*, *60*, 34–47. https://doi.org/10.1016/j.socnet.2019.04.004

# Appendix B: Additional Tables

**Table B.1** Multilevel Random Intercepts Model Predicting Individual Peer Rejection Score using Individual and Classroom-level Characteristics (sample restricted to same classrooms as in metaregression; N=3,045)

|  | Model 1 | Model 2 | Model 3 |
| --- | --- | --- | --- |
| Individual characteristics |  |  |  |
| Migration background (Swedish origin) | -0.022** | -0.038** | -0.039** |
|  | (0.007) | (0.007) | (0.008) |
| Gender (Girl) | -0.021** | -0.022** | -0.022** |
|  | (0.006) | (0.006) | (0.006) |
| Household income (in 100 000 SEK) | -0.003** | -0.005** | -0.005** |
|  | (0.001) | (0.001) | (0.001) |
| Parental education (in years) | -0.002+ | -0.003** | -0.003** |
|  | (0.001) | (0.001) | (0.001) |
| Cognitive test score | -0.004** | -0.004** | -0.004** |
|  | (0.001) | (0.001) | (0.001) |
| Classroom characteristics |  |  |  |
| Share of Swedish origin |  | 0.065** | 0.080** |
|  |  | (0.016) | (0.022) |
| Share of girls |  | 0.056* | 0.138** |
|  |  | (0.024) | (0.038) |
| Mean household income |  | 0.003 | 0.003 |
|  |  | (0.004) | (0.004) |
| Mean years of parental education |  | 0.004 | 0.004 |
|  |  | (0.004) | (0.004) |
| Mean cognitive test score |  | 0.001 | 0.001 |
|  |  | (0.002) | (0.002) |
| Cross-level interactions |  |  |  |
| Swedish origin*Share of Swedish origin |  |  | -0.037 |
|  |  |  | (0.025) |
| Gender*Share of girls |  |  | -0.146** |
|  |  |  | (0.049) |
| Household income*Mean hh. inc |  |  | 0.001 |
|  |  |  | (0.000) |
| Parental education*mean yrs par. educ. |  |  | -0.001 |
|  |  |  | (0.001) |
| Cognitive test score*Mean cognitive test score |  |  | -0.001* |
|  |  |  | (0.000) |
| Constant | 0.125** | 0.137** | 0.145** |
|  | (0.007) | (0.007) | (0.008) |

Note: + p< 0.10, * p<0.05, ** p<0.01
